# Integrative Identification of *Arabidopsis* Mitochondrial Proteome and Its Function Exploitation through Protein Interaction Network

**DOI:** 10.1371/journal.pone.0016022

**Published:** 2011-01-31

**Authors:** Jian Cui, Jinghua Liu, Yuhua Li, Tieliu Shi

**Affiliations:** 1 College of Life Sciences, Center for Bioinformatics and Institute of Biomedical Sciences, East China Normal University, Shanghai, China; 2 College of Life Sciences, Northeast Forestry University, Harbin, Heilongjiang, China; 3 Southern Medical University, Guangzhou, Guangdong, China; 4 Shanghai Information Center for Life Sciences, Chinese Academy of Sciences, Shanghai, China; 5 Daqing Institute of Biotechnology, Northeast Forestry University, Daqing, Heilongjiang, China; University of South Florida College of Medicine, United States of America

## Abstract

Mitochondria are major players on the production of energy, and host several key reactions involved in basic metabolism and biosynthesis of essential molecules. Currently, the majority of nucleus-encoded mitochondrial proteins are unknown even for model plant *Arabidopsis*. We reported a computational framework for predicting *Arabidopsis* mitochondrial proteins based on a probabilistic model, called Naive Bayesian Network, which integrates disparate genomic data generated from eight bioinformatics tools, multiple orthologous mappings, protein domain properties and co-expression patterns using 1,027 microarray profiles. Through this approach, we predicted 2,311 candidate mitochondrial proteins with 84.67% accuracy and 2.53% FPR performances. Together with those experimental confirmed proteins, 2,585 mitochondria proteins (named CoreMitoP) were identified, we explored those proteins with unknown functions based on protein-protein interaction network (PIN) and annotated novel functions for 26.65% CoreMitoP proteins. Moreover, we found newly predicted mitochondrial proteins embedded in particular subnetworks of the PIN, mainly functioning in response to diverse environmental stresses, like salt, draught, cold, and wound etc. Candidate mitochondrial proteins involved in those physiological acitivites provide useful targets for further investigation. Assigned functions also provide comprehensive information for *Arabidopsis* mitochondrial proteome.

## Introduction

Mitochondrion is a semi-autonomous organelle controlled by two genomes - its own and that of the nucleus. The plant mitochondrial proteome might contain as many as 2,000–3,000 different gene products, but only a few proteins, rRNAs and tRNAs are encoded by the *Arabidopsis thaliana* mitochondrial genome. Therefore the majority of mitochondrial proteins are encoded by the nuclear DNA, coordinated by the gene expression between the two genomes precisely. Besides the production of ATP in the process of oxidative phosphorylation and the tricarboxylic acid (TCA) cycle, mitochondria also play pivot roles in signal transduction processes and pathway of communication between mitochondria and the nucleus, produce the biosynthetic precursors , such as the synthesis of nucleotides, amino acids, lipids, and vitamins [1],[2],[3], and actively participate in regulation of programmed cell death (PCD) [4], [5], [6]. In addition, they are also involved in the execution of adaptive response in response to increased oxidative stress levels, aspects of cytoplasmic male sterility and behaviors of ionic homeostasis as well [7].

*Arabidopsis thaliana* genome has been sequenced by the *Arabidopsis* Genome Initiative (AGI) [8] and scientists have experimentally verified about 1,300 distinct *Arabidopsis thaliana* proteins, which are distributed among different compartments, with most of the proteins localized to mitochondria (36%), followed by other three major compartments: nucleus (28%), plastid (17%), and cytosol (13.3%), respectively [9]. However, a majority of mitochondrial proteins and their functions are still poorly understood. Curation and analysis of the *Arabidopsis* genome by The Institute for Genomic Research (TIGR) [10] and The *Arabidopsis* Information Resource (TAIR) [11] have generated an annotated genome with high quality, but verified localization of proteins in *Arabidopsis* mitochondria is not much. Meanwhile, *Arabidopsis* mitochondria proteins deposited in SwissProt are also limited (∼227 proteins).

This situation stimulates the development of subcellular proteomics, a strategy that provides encouraging advances towards to the goal that directly contributes to protein annotations, since detecting the protein subcellular localizations is an important step to understand protein function and cell behaviors. As one of major advanced technologies in post-genomic biology, subcellular proteomics has higher capability in discovering protein functions systematically from spatial and time scales.

In order to identify protein subcelluar localization, purification methods, such as density gradient centrifugation [12], [13], [14], [15], [16], [17], [18], immunoisolation [17], and free-flow electrophoresis [19], have been developed and shown improved effects in identification of more specific subcellular proteins. Recently, many other novel experimental tools and analysis strategies have been introduced in advancing plant mitochondrial proteomics research. Random and directed epitope-tagging techniques have been used as proteome-scale analysis in yeast [20]. Two-dimensional electrophoresis (2D-PAGE) studies have defined the size of *Arabidopsis* mitochondrial proteome in two systematical studies [21], [22]. Additionally, the combination of three different gel electrophoresis procedures (three-dimensional gel electrophoresis) has been also used for subdivision of *Arabidopsis* mitochondrial proteome [23]. Meanwhile, various mass spectrometry techniques become the most frequently employed approaches to identify the components of mitochondrial proteomes of plants, due to their sensitive, selective, and relatively unambiguous nature. A direct sample analysis by liquid chromatography and tandem MS (LC-MS/MS) on Arabidopsis mitochondrial proteomics have obtained a set of ∼400 proteins with 20% of them unknown function [24]. An alternative strategy to subcellular proteomics is GFP technique which provides a direct way to confirm subcellular location for a protein. The GFP gene is frequently used as a reporter of expression and is normally in frame linked to the studied gene [18]. In cells where the analyzed gene is expressed, and the tagged protein is produced, GFP is generated at the same time. Then, the GFP can be observed under fluorescence microscopy, which is the indicator for the expression of the target gene and the location of its protein. Analysis of such time lapse movies has redefined the understanding of many biological processes including protein folding, protein transport, and protein sub localization [25]. High through-put GFP screening of protein subdivision has already been on the way for *Arabidopsis* [26], [27], [28]. The data on subcellular localizations of *Arabidopsis* mitochondrial proteins based on GFP image and MS/MS can be queried in SUBA database [29].

On the other hand, many bioinformatics tools, such as TargetP[30], MitoProt [15], iPSORT [31], WoLF PSORT[32], and Predotar [33], *etc.*, have been developed for predicting the protein subcellular locations within cells. The principles of those tools are usually based on identification of sequence features from amino acid compositions by various machine learning algorithms, including neural networks [34], Hidden Markov Models (HMMs) [35], Support Vector Machines (SVMs) [12], [36] and Nearest Neighbors [37], [38], *etc*.

Integrating information from disparate types of genomic data to understand cellular functions have been emphasized recently [39], [40], [41], [42]. Each emerged approach may have its own bias on mitochondrial localization detection, be a lack of evaluations with a common benchmark and finally cause more confusing interpretations on mass published datasets by direct comparisons. The aim of our study is to identify more comprehensive and reliable genes encoding the mitochondrial proteins and finally analyze their biological functions. Firstly, we describe an application of integrative genomic-scale methodology to identify a set of reliable nuclear-encoded mitochondrial proteins in plant *Arabidopsis*. This approach not only systematically expands the catalog of mitochondrial proteins in the model plant *Arabidopsis*, but also gives a systematical assessment on fourteen genomic-scale predictors for identifying mitochondria proteome. Those predictive features come from gene co-expression profiles, protein domains, orthologous group mappings and some popular programs. A statistical approach, named Naïve Bayesian Network, integrated such disperse genomic-scale predictors and gained a more comprehensive and reliable set of core mitochondrial proteins, named CoreMitoP, by joining experimentally verified ones and excluding false predicted ones. Particularly, as for those proteins in CoreMitoP with unknown functions, we applied a network-based approach to search functionality of newly predicted proteins according to its positions in protein interaction network (PIN), considering the functionality of its direct and indirect neighborhoods.

## Methods

### *Arabidopsis* datasets and Training Sets for assessing localization predictions

30,480 peptide sequences *of Arabidopsis* were downloaded from TAIR [11]. Mitochondrial genome and chloroplast genome encoded proteins are excluded in this analysis. IPI database (EBI) (www.ebi.ac.uk/IPI/) was adopted to provide a convenient identifier conversion among SwissProt, NCBI and other reference database. Uniformly, we used plant standard AGI symbols, the same as TAIR.

#### GSP_mito_ dataset

Gold-standard positives or gold-standard mitochondrial proteins possess experimentally observed evidences and are recorded in SUBA database [29]. We collected total 894 experimental verified mitochondrial proteins encoded by nuclear genome from five resources, including GFP assay (151 proteins), MS/MS assay (501), TAIR(415), AmiGO (97) and UniProt (112). To guarantee training accuracy, the proteins with MS records that target to nonmitochondria compartments have been removed and 806 proteins were finally determined as **GSP_mito_** for training (**Data S5**). Otherwise, **GSN_∼mito_ dataset:** Gold-standard non-mitochondrial proteins **GSN_∼mito_** is generated from SwissProt (**Data S5**). SwissProt contains 2,374 clearly well defined non-mitochondrial proteins, including proteins localized at cell plate, cytoskeleton, cytosol, endoplasmic reticulum, extra cellular, Golgi, nucleus, peroxisome, plasma membrane, plastid and vacuole. Then, we mapped SwissProt accession numbers to AGI symbols, and got 1,464 nonmitochondrial proteins and ensured that there is no intersection with GSP_mito_.

### Fourteen predictors for Bayesian Network integration

s1 refers to predictions by MitoProtII [15] that uses discriminant analysis to indicates the presence or absence of N-terminal mitochondrial targeting sequence. s2 refers to predictions by iPSORT (http://hc.ims.u-tokyo.ac.jp/iPSORT/), with plant protein option selected. As the first comprehensive localization prediction method to be developed for plant, iPSORT can reflect various characteristics of the sequence and give out final predictive conclusions through the k-nearest-neighbor classification technique [31]. s3 is computed by TargetP software (http://www.cbs.dtu.dk/services/TargetP), which applies neural network [30], [34] to classify proteins into two classes of mitochondrial and non-mitochondrial types. Default parameters are chosen for plant option. s5 feature is conducted by Predotar on web server (http://www.inra.fr/Internet/Produits/Predotar). Predotar is particularly good at distinguishing mitochondrial and plastid targeting sequences as previously reported [33]. Here, Predotar version 1.03 was chosen to be applied on *Arabidopsis* proteome with plant sequences selected;

s6 is computed by protein domain method, which indicates the presence or absence the Pfam domain occurrence patterns and the amino acid compositional differences that the presence of protein domains found to be exclusively mitochondrial, exclusively non-mitochondrial or shared based on the SwissProt annotation of all eukaryotic sequences. We attained 3,839 proteins with SwissProt identifiers corresponding to confidences > = 85%. Then we mapped these proteins to 2,614 *Arabidopsis* proteins with AGI identifiers.

s7 comes from loctree, a hierarchical system combining support vector machines (SVMs) and other prediction methods. LOCtree predicts the subcellular compartment of a protein by mimicking the mechanism of cellular sorting and exploiting a variety of sequence and predicted structural features in its input [43].

s8 is provided by WoLF PSORT (http://wolfpsort.seq.cbrc.jp). Its prediction accuracy is updated by applying feature selection and simple k nearest neighbor classifier for classification [32] with plant option and prediction score > = 4 that are used to determine localization (the top prediction < = 3 were designated as unknown). S9 is generated by MultiLoc, a tool with the intention to predict all of the main subcellular location [44]. Several additional features have been incorporated in order to facilitate for the extended number of localizations to be discriminated. Furthermore, a subprediction method (SVMSA) for detecting signal anchors (SAs) is used.

Ideally, localization is an evolutionarily conserved trait, homologues in different organisms tend to localize at the same sub-compartment in a cell [45]. Phylogenetic studies of the *S. cerevisiae* and *C. elegans* mitochondrial proteome have shown a complex evolutionary scenario[46]. This allows the transfer of function or annotation based on sequence-similarity; if a query protein displays significant similarity to a known (or confidently predicted) mitochondrial protein, the chance is that the query sequence is also a mitochondrial protein. Mitochondria are commonly known as the result of the endosysmbiosis by an ancestral cell, *Rickettsia prowazekii* [47]. It could be expected that mitochondria utilize the machinery inherited from their bacterial progenitor. Thus, s10 is obtained by ancestry transfer method that measures the *Arabidopsis* sequence similarity to *Rickettsia prowazekii* proteomes, the closest living bacterial relative of plant mitochondria. *Rickettsia prowazekii* totally contains 835 proteins in NCBI. Through BLASTP program detection, *Arabidopsis* has 1,960 *Rickettsia prowazekii* homologs according to the filtering criterion (E< = E-15, coverage > = 85%), as *Arabidopsis* mitochondrial proteins. Otherwise, Human orthologs (s11), Mouse orthologs (s12) andYeast orthologs (s13) indicate the existence or absence of *Arabidopsis* orthologs in mitochondrial proteomes of *Homo sapiens*, *Mus musculus* and *Saccharomyces cerevisiae* by experimental approaches or manual mining literatures. Using the Mitochondrial Proteome Database (http://141.39.186.157:8080/mitop2/), which (2006 version, 2006-11-07) lists 521 yeast mitochondrial proteins, 1,019 human mitochondrial proteins and 731 mouse mitochondrial proteins manually annotated by the MitoP2 team according to the published experimental data. We identify potential *Arabidopsis* orthologs through orthologs transfer between species. Eukaryotic Ortholog Groups were downloaded from the Inparanoid eukaryotic ortholog database (http://inparanoid.sbc.su.se/). It uses BLAST scores to measure relation of proteins and detect complex orthologous relationships between species. An *Arabidopsis* protein was assigned a categorical score of 1 if *Arabidopsis* orthologs of the human, mouse or yeast mitochondrial protein exists or assigned a score of 0 otherwise. Through proper identifier conversion between databases, we got 659 *Arabidopsis* proteins, the genes of which have orthologous relationship in 1,019 human mitochondrial reference set with experimental evidence collected from MitoP2. Similarly, unique 705 *Arabidopsis* orthologs in mouse mitochondrial proteins and 652 *Arabidopsis* orthologs in yeast mitochondrial proteins were identified.

s14 is obtained through Gene coexpression analysis. Iintegrative gene expression profiles are downloaded from TAIR FTP site (ftp://ftp.arabidopsis.org/home/tair/Microarrays/) (See Data S3) which are designed through an international effort to develop a gene expression atlas of *Arabidopsis* which has been under way since fall 2003. This project, called AtGenExpress, provides the *Arabidopsis* community with access to a large set of Affymetrix Microarray data. These comprehensive datasets focus on different tissues and different developmental stages and treatments (environment stress or mutants). The datasets were preprocessed and normalized by using RMA method [48] embedded in Affy package downloaded from Bioconductor (www.bioconductor.org/), and then we utilized N50 metric strategy for mitochondrial protein prediction [49]. Since the neighborhood metric can score each gene's coexpression with known mitochondrial genes, the principle of N50 metric is to count each gene's coexpression with known mitochondrial genes. The number of GSP_mito_ genes within a gene's 50 closest neighbors (Euclidean distance) was generated for each profile. We obtained twenty-four N50 count vectors from total 1,027 microarrays for 22,180 probesets. Then, we used decision tree applied in Weka (http://www.cs.waikato.ac.nz/ml/weka/) to train such twenty four N50 vectors and conducted predictions for mitochondrial proteins with J48 (C4.5) algorithm ([weka.classifiers.trees.J48 -C 0.25 -M 2] (Test mode: 10-fold cross-validation). Consequently, we obtained 1,727 putative *Arabidopsis* mitochondrial proteins on the whole genome-wide scale with 88.19% correctly classified instances.

### Naïve Bayesian Network

Bayesian networks have several advantages for our integration task here: They allow for combining different types of data (i.e., numerical and categorical), converting them to a common probabilistic framework. Bayesian networks are readily interpretable as they represent conditional probability relationships among information sources and formal representation of such relationships between features. The Bayesian network should ideally be independent from the data sources serving as evidence, sufficiently large for reliable statistics [50], [51], [52].

So, we proposed this approach for integrating mitochondrial protein information for *Arabidopsis*. The basic idea is to assess each source of evidence for protein subcellular localizations by comparing it against samples of known (“gold-standards”) positives (**GSP_mito_**) and negatives (**GSP∼_mito_**), yielding a statistical reliability. We predicted the chance of possible mitochondrial localization for every protein by combining each independent evidence source according to its reliability.

Conditional independence means that the information in the N datasets is independent given that a protein is either positive (GSP_mito_) or negative (GSN_∼mito_). Bayesian networks are a representation of the joint probability distribution among multiple variables (which could be datasets or information sources). Formally, they can be described as follows: We define ‘positive’ proteins as GSP_mito_ that are located in the mitochondria and ‘negative’ proteins as GSN_∼mito_ that are located in the non-mitochondrial organelles. Given the number of positives among the total number of proteins, the ‘prior’ odds of finding a positive are: 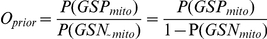



In contrast, the ‘posterior’ odds are the odds of finding a positive after we consider N datasets with values s1 … s_N_:


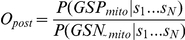
, in which numerator is 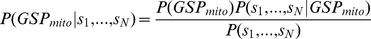


(The terms ‘prior’ and ‘posterior’ refer to the situation before and after knowing the information in the N datasets, e.g., s_1_… s_N_) Then, we reformulate the model using Bayes' theorem to make the joint probability in the numerator more tractable. then the ‘posterior’ odds is derived as:



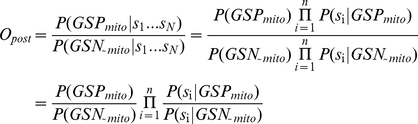



It is mitochondrial protein if 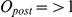, otherwise it is classified as non-mitochondrial protein.

Since 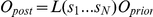, in which, 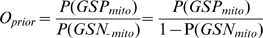, L as a likelihood ratio that relates prior and posterior odds according to Bayes' rule can be simplified to


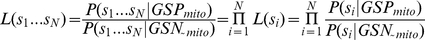
, assuming that the features are independent.

In our binary classification practice, 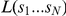 can be computed from all model (s_1_,…,s_n_) parameters (e.g., class priors and feature probability distributions) approximated with relative frequencies from the training set (e.g., 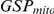 and 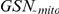), such as: 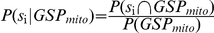 and 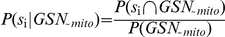.

Here, we defined ArathMitoP set as integrated mitochondrial protein set generated from 14 predictors by using Naïve Bayes Network and defined the ArathMitoP set score for a protein as logLR. The likelihood ratio (LR) indicates a correlation between the feature and the class. The greater the LR is, the more reliable the performance of the classification is.

### Prediction performance metrics

For a particular classifier, various standard performance metrics can be summarized based on the confusion matrix to compare model prediction performance. The Confusion Matrix is represented with the form in Table 1. Sensitivity is a measure of actual positives correctly identified and the specificity measures the proportion of negatives correctly identified: The false discovery rate (FDR) is the proportion of all predictions that are false, estimated from gold-standard negative and positive training sets (e.g. GSP_mito_ or GSN_∼mito_).

**Table 1:** Confusion matrix used for evaluating predictive performance.

Total Samples (TS)	Actual Positives (AP)	Actual Negatives (AN)
Predicted Positives (PP)	True Positives (TP)	False Positives (FP)
Predicted Negatives (PN)	False Negatives (FN)	True Negatives (TN)



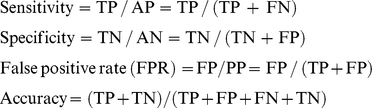



There is a trade-off between sensitivity and specificity, making models difficult to compare on the basis of these performance metrics. In contrast, such measures as accuracy, the proportion of correct predictions, and the ROC (receive operating characteristic) curve enable a single parameter comparison of performance of binary classification models. The ROC curve can provide a graphical representation of the relationship between the true-positive and false-positive prediction rate of a model, and evaluate the metrics of its performance.

The greater the sensitivity value is at high specificity values (i.e. high y-axis values at low x-axis values), the better the model is. Clearly, the ROC curve for a good classifier will be as close as possible to the upper-left corner of the chart,that is where we have the highest number of true positives and at the same time the smallest number of false positives. Some authors emphasize the importance of quality (higher accuracy amounts to more correct predictions) over quantity (a higher number of predictions), we used ROCR package in R language environment to accomplish this computation [53] and gave out a proper logL threshold for both of the two metrics (e.g. FPR and sensitivity).

### Inferring Protein functionality using PIN-based approach

The prediction for protein functionality is applied by evaluating the similarity of topological properties between itself and its level-1(direct) or level-2 (indirect) neighbors in protein interaction networks (PIN) [54]. Following the original method, the functional similarity of two proteins, u and v, is evaluated using the Czekanowski-Dice distance (CD-Distance). The CD-distance between two proteins u and v is given by 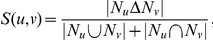
where Np refers to the set that contains p and its level-1 neighbours and 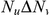 refers to the symmetric difference between two sets 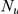 and 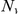; Then, we only considered the transitive functional association model. That is if protein u is similar to protein w, and protein w is similar to protein v, proteins u and v may show some degree of similarity. We used this transitive functional association to estimate the functional similarity between u and v by the product of the functional similarity between u and w, and that between w and v. 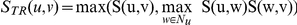



Using the functional similarity as measure, we applied averaging method to predict the function of a protein based on the functions of the level-1 and level-2 neighbours. The probability that a protein p has a function x is estimated by



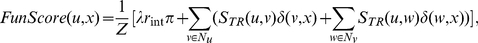


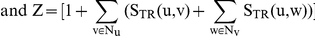




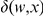
 = 1 if p has function x, 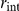 is defined as the fraction of all interaction pairs that share some function; 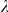 = 1; 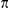 is the frequency of function x in annotated proteins representing the contribution of background frequency to the score; Z is used for normalizing the probability FunScore(u,x).

For *Arabidopsis*, 28,091 reliable protein–protein interactions were downloaded from updated AtPID [55]. Currently available functional annotation for *Arabidopsis* genome come from GO (www.geneontology.org/) and were formatted as form of 46,696 protein-function pairs containing 1,381 functionality for total 18,039 proteins. Particularly, the Arabidopsis function categories are confined to level 4 and 5 according to GO tree structure. This restriction is needed for capturing relatively higher frequencies for those specific and interested function categories, because superabundant categories like (1) ubiquitous function categories with very high frequency or (2) very specific function categories with very low frequencies can both cause global function frequency very low and ubiquitous or very specific functions are not necessary to predict here. Otherwise, another criterion to choose or define function categories is that most of proteins are recorded within such GO levels (14,246 proteins for Level 4, and 13,406 proteins for Level 5)(**Data S6**).

By searching PIN structure and known protein function categories, we evaluated each function x for each unknown protein in CoreMitoP (p) based on its frequency of the protein's neighborhoods, FunScore(p,x). Meanwhile, to validate the effectiveness of the method for function enrichment, the proteins in PIN with known functional categories were applied by the same PIN-based prediction procedure and we obtained corresponding 4,621 protein-function pairs containing total 2,274 proteins and 221 relative functional categories (GO terms). The FunScore for proteins with the real function categories are all above 0.03 by conducting network-based protein function prediction, and FunScore> = 0.03 provides a preliminary filtration for significant protein-function pairs (**Data S6**). Further, 10,000 permutations for global protein functional categories were performed and null distribution of FunScore for each protein-function pair computed based on the same procedure of PIN-based method can be obtained for evaluating its p value.

## Results

### Fourteen predictors for mitochondrial localization

**Figure 1:**
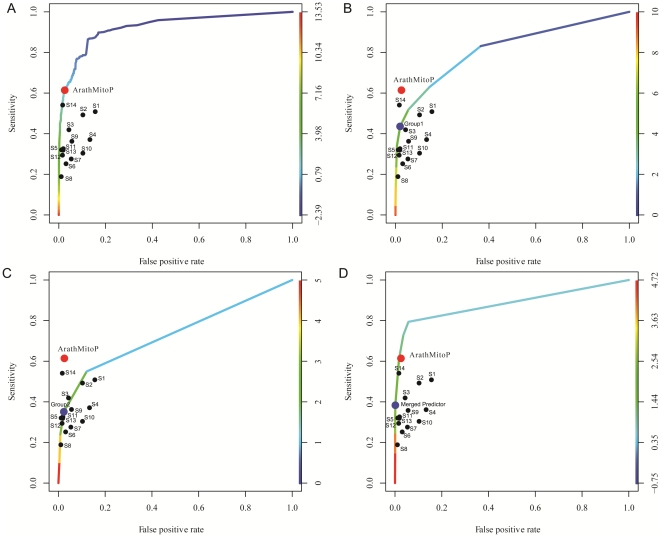
Performance evaluation metrics of mitochondrial prediction methods. (A) Sensitivity and false positive rate of *Arabidopsis* mitochondrial prediction methods. Using training data sets of 894 known Arabidopsis mitochondrial proteins (GSP_mito_) and 1,464 non-mitochondrial proteins(GSN_∼mito_), we estimated the sensitivity (percentage of GSP_mito_ correctly predicted) and false positive rate of each prediction method. The accuracies of the thirteen individual data sets (s1∼s14) are shown at specific thresholds, while ArathMitoP set is drawn as a colorful ROC curve and the chosen threshold is noted with a red circle, at which we can obtain a good balance between the two performance metrics (i.e. FPR and sensitivity)…black circles indicate other s1–s14 predictive powers. (B). ROC curve of predictive powers for indirectly merged predictors, s1–s9. Blue circle indicates the threshold for indirectly merged s1–s9 predictors (named Group1); red circle indicates the power for ArathMitoP predictions, black circles indicate other s1–s14 predictive powers. (C). ROC curve of predictive powers for indirectly merged predictors, s10–s13; blue circle indicates the threshold for indirectly merged s10–s13 predictors (named Group2); red circle indicates the power for ArathMitoP predictions, black circles indicate s1–s14 predictive powers. (D). ROC curve of predictive powers for integrated three groups generated under the indirectly merge strategy. blue circle indicates the threshold for merged integration (named Merged Predictor); red circle indicates the power for ArathMitoP predictions, black circles indicate s1–s14 predictive powers.

We constructed fourteen genomic-wide predictors for *Arabidopsis* before integration (indexed by s1,…,s14), each of the predictors is currently available or extensively analyzed on the whole 30,480 peptide sequences from TAIR (June 19, 2009 release) that together provides the most comprehensive *Arabidopsis* genome annotation (**Data S1**). Firstly, we applied nine existent bioinformatics approaches, they are: MitoProtII (s1), Ipsort (s2), TargetP (s3), SubLoc (s4), Predotar (s5), Protein domain (MitoPred) (s6), Loctree (s7), WoLF Psort (s8) and Multiloc(s9), all of which indicate the presence or absence of an N-terminal mitochondrial targeting sequence and other sequence characteristics that directs protein importing into the mitochondria by utilizing multiple chemical and physical properties of proteins for classification of mitochondrial components through various machine learning algorithms. These nine predictors provide genomic-wide predictions for mitochondrial protein localization, which containing 1,000∼5,000 predictive proteins with 70% average accuracy tested on the gold standard datasets (Figure 1A, Figure 1B, and Table 2). Particularly, four programs, including (s1)MitoProtII, (s2)Ipsort, (s3)TargetP, and (s5)Predotar, share the most with 615 predictions in common, those overlapping predicted proteins are included in the final core mitochondrial set (CoreMitoP).

**Table 2:** Fourteen genome-scale data sets used to predict mitochondrial localization.

Predictioin methods	Description for the genome-scale data set	Proteins predicted	Accuracy	Sensitivity	Specificity	FPR	FDR
(s1)MitoProtII	predictions by MitoProtII (MG Claros, etc.1996)	4,222	72.51%	50.87%	84.43%	15.57%	35.74%
(s2)Ipsort	predictions by Ipsort (Nakai K etc.1999)	4,972	75.33%	49.26%	89.69%	10.31%	27.55%
(s3)TargetP	predictions by TargetP (Olof Emanuelsson, etc.2000)	3,179	76.61%	41.94%	95.70%	4.30%	15.71%
(s4)SubLoc	SVM method based on amino acid composition (Sujun Hua,etc 2001)	3,765	69.07%	37.10%	86.68%	13.32%	39.47%
(s5)Predotar	predictions by Predotar (Small, I.,etc.2004)	1,142	75.11%	32.01%	98.84%	1.16%	6.18%
(s6)Protein domain	Pfam domain Method by MitoPred Algorithm (Guda C etc.2004)	2,614	71.45%	25.19%	96.93%	3.07%	18.15%
(s7)Loctree	A novel system of SVMs (Rajesh Nair and Burkhard Rost, 2005)	3,320	70.84%	27.54%	94.67%	5.33%	26.00%
(s8)WoLF Psort	predictions by WoLF Psort (Paul Horton etc.2006)	1,036	70.53%	18.86%	98.98%	1.02%	8.98%
(s9)Multiloc	A SVM-based approach, which integrates N-terminal targeting sequences, sequence motifs, and amino acid composition (Annette Höglund, etc., 2006)	3,284	73.74%	36.23%	94.40%	5.60%	21.93%
(s10)Ancestry	R. prowazekii homologs (Andersson SG, 1998) The genome sequence of Rickettsia prowazekii and the origin of mitochondria. Nature. 1998 Nov 12;396(6707):109–10.	1,960	68.63%	30.40%	89.69%	10.31%	38.13%
(s11)Human ortholog	orthologs of Homo sapiens mitochondrial	659	74.85%	32.63%	98.09%	1.91%	9.62%
(s12)Mouse ortholog	orthologs of Mus musculus mitochondrial	705	73.92%	29.40%	98.43%	1.57%	8.85%
(s13)Yeast ortholog	orthologs of S. cerevisiae mitochondrial	652	74.63%	31.89%	98.16%	1.84%	9.51%
(s14)Coexpression	Coexpression with known mitochondrial genes in Arabidopsis	1,727	82.64%	54.09%	98.36%	1.64%	5.22%
ArathMitoP set	Integration by Naïve Bays Network (LR>1.37)	2,311	84.67%	61.41%	97.47%	2.53%	6.95%

Fourteen individual predictors and an integrated predictor (named ArathMitoP set) were conducted to predict mitochondrial localization of all 30,480 Arabidopsis proteins in TAIR. Performance evaluation metrics on genome-wide, such as sensitivity, specificity, false positive rate and false discovery rate, were estimated based on large gold standard training data. (FPR: false positive rate; FDR: False discovery rate).

Secondly, we used evolutionary conserved sequence features to predict mitochondrial proteins. The ancestry predictor (s10) measured the presence or absence of *Arabidopsis* mitochondrial homologs in *Rickettsia prowazekii* and returned 1,960 sequences from BLAST program with E< = 1e-15, consequently. Meanwhile, other three predictors (s11–s13) generated from eukaryotes ortholog mappings have also capabilities to assess *Arabidopsis* mitochondrial proteome. Orthologs were determined through high-stringency sequence homology matching using the program INPARANOID [56].This program searches for high stringency orthologous clusters between two protein sets, providing clusters of paralogs within species and orthologs across species. The potential *Arabidopsis* mitochondrial proteins are matched putative orthologs from human, mouse and yeast proteins those all localize at mitochondria and are verified experimentally. *Arabidopsis* homologs from mitochondrial proteome of human, mouse and yeast are considered as complementary phylogenetic hints., In consequence, the four predictors (s10–s13) based on phylogenetic studies are independently integrated.

Gene expression profiles were also used as an evidence for inferring protein subcellular localization because genes with similar expression patterns or relatively high correlation coefficient are potentially within the same cellular compartments or pertaining to relational functionality [57]. The coexpression method (s14) measures transcriptional coexpression with known mitochondrial genes, using a genomic-scale RNA expression data across diverse tissues and conditions. We collected twenty-four comprehensive expression datasets from AtGenExpress [58] (**Data S2**) designed for *Arabidopsis* and applied a neighborhood metric (Materials and Methods), N50 metric, to score each gene's coexpression strength with *Arabidopsis* mitochondrial genes (golden-standard positive dataset) and obtained 1,727 predictions with 82.64% accuracy.

### Predictive power of individual predictor and the statistical integration

To improve prediction accuracy, we integrated the results obtained from the 14 genome-wide predictors using a Naïve Bayesian Network, and generated a new catalog of putative mitochondrial proteins (called ArathMitoP set), statistically. Correspondingly, the 14 predictors were considered individually. We evaluated the performance of each method with large ‘gold standard’ training sets: 894 mitochondrial proteins (GSP_mito_) from SUBA and 1,464 nonmitochondrial proteins (GSN_∼mito_) annotated with localizations at other cellular compartments (Materials and Methods, **Data S3**). Specifically, for each *Arabidopsis* gene product p, we assigned a score S_i_ (p) (i = 1,…,14), as a likelihood of mitochondrial localization by comparing performance on GSP_mito_ with performance on GSN_∼mito_ at a range of Likelihood Ratio (LR), and gained ArathMitoP LR for each protein by summing the each log-LR of the fourteen predictors (Materials and Methods, Data S3). Using a conservative threshold of 1.37, obtained ArathMitoP set contains 2,311 potential mitochondrial proteins properly predicted with 82.74% accuracy and 2.66% false positive rate (**Fig. 1A** **, and** **Table 2**). It also generated 1,029 proteins not in the training dataset GSP_mito_. Meanwhile, predictive performances of the 14 predictors were also evaluated against the same training datasets (GSP_mito_ and GSN_∼mito_) and we found that most of the predictors by bioinformatics tools (s1–s9) and predictors based on phylogenetic studies (s10–s13) perform at ∼70% accuracy, and less than 50% specificity. Interestingly, the predictor s14 by coexpression method achieves 82.64% accuracy and 1.64% FPR. So, integrated ArathMitoP set improves the quality and relative quantity of mitochondrial proteins with better predictive performance.

Alternatively, because some predictors have certain similarity in training, we also assessed whether the minified predictors may affect the predictive power. We used two strategies to surrogate whether different types of merged predictors have great variance on predictive power: (1) directly merge predictors; (2) indirectly merge predictors. Firstly, we directly merge s1–s9 predictors into Group 1, which contains total 13,312 proteins; and merge s10–s13 proteins into Group2, which containing 2.778 proteins. Remained s14 is defined as Group3. Then, we used the same workflow of Bayesian network to predict results. We obtained 1,052 predictions above LR threshold with 80.57% accuracy, 46.8% sensitivity and 3.32% FDR. All three metrics (accuracy, sensitivity and FDR (false discovery rate)) of integration power from minify groups are all obviously lower than performances of the former universal integration. Secondly, we used indirectly merge strategy, which means we count the record times by predictors within a grouped predictors (e.g Group1 = {s1,…s9}, Group2 = {s10…s13}, and Group = s14) and then conduct training of such records for each protein on GSP and GSN and choose an appropriate record threshold, and finally use the threshold to predict results. For Group1 and Group2, we chose 4 and 2 records as their thresholds, respectively. Then, Group1 predicted 2,064 proteins with 43.55% sensitivity, 1.98% FPR, and 78.68% accuracy and Group2 predicted 706 proteins with 35.11% sensitivity, 2.32% FPR and 75.46% accuracy. Their predictive powers are shown in Fig 1B and C. Latsly, we used the same Bayesian network to integrate such three groups and obtain 515 proteins above the threshold. Its integration performances is illustrated in Fig 1D. Obviously, multiple types of merged integration powers are limited compared with original universal integration. Thus, we took original integrative results from all 14 predictors for following analysis.

The number of proteins uniquely predicted as mitochondrial by each of the nine predictors (s1–s9) of bioinformatics tools ranged from 1,000 to 5,000 (**Table 2**). Overall, these methods generally predicted about 18% to 49% known mitochondrial proteins in GSP_mito_. However, large numbers of predictions cause more confusion and noises, while low-confidence predictions can be partially attributed to the contradictions between sensitivity and specificity of each predictor's performance. Some researchers may emphasize on the importance of correct predictions that amounts to the sensitivity metric, while others pay more attention to a high number of predictions that amounts to the specificity metric. The nine programs can not obtain satisfied balances from the testing against GSP_mito_ and GSN_∼mito_ datasets. TargetP achieves 95.70% specificity, but only 41.94% sensitivity, which gives rise to a 4.3% false positive predictive rate. MitoProtII(s1), iPSORT(s2), Predotar(s5), WoLF PSORT(s8) and MitoPred (s6) [59] predictive power are also restricted from views of specificity, sensitivity or FDR requirements (**Table 2**).

To further assess the performances of those bioinformatics programs in details, we considered the overlapping predictions by the five normally used predictor programs (s1, s2, s3, s5 and s8). Positive and negative prediction numbers were used to describe comparisons among the predictor programs. Positive prediction numbers indicate the number of proteins predicted as mitochondrial only by this predictor program compared with others. Negative prediction numbers indicate the number of proteins predicted as mitochondrial by all other predictor programs but not by this predictor program. As the results, TargetP(s3) identified 3,179 proteins, 1,645 of which (51.75%) are included in ArathMitoP set and 338 proteins in GSP_mito_ (achieves 41.94% sensitivity) are included in s3. That means 666 proteins in ArathMitoP set have no recognizable targeting signals that show the limitation for certain signal-based methods.

**Figure 2:**
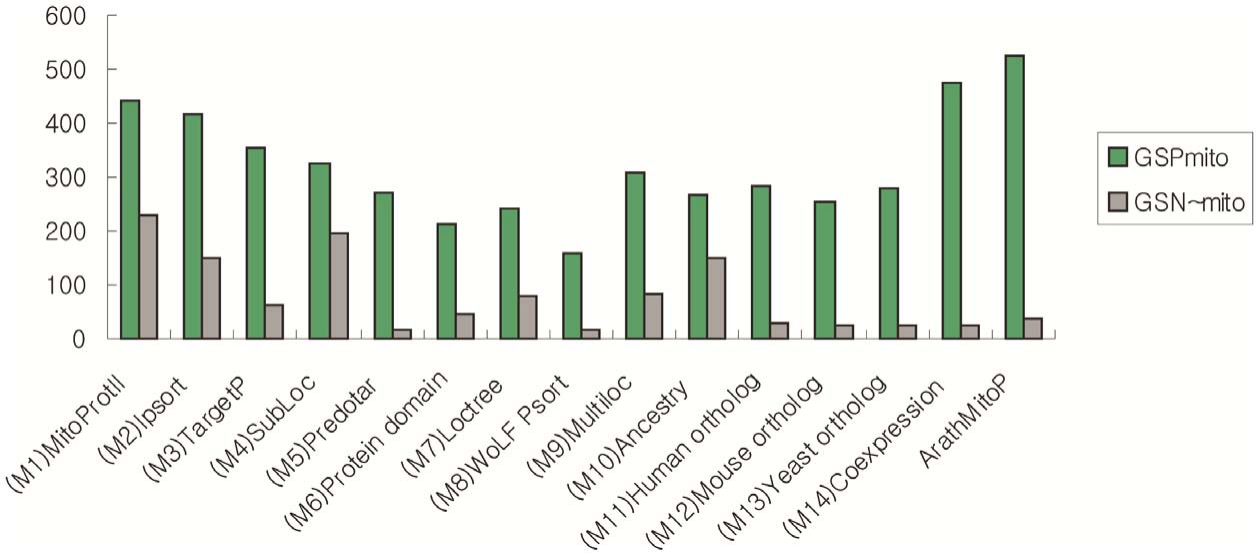
The coverage of 14 individual predictors and the ArathMitoP set with GSP_mito_ and GSN_∼mito_. The number of overlapped proteins between si (i = 1,…,14) and training data sets is shown individually. Green bars indicate the intersection between si and GSP_mito,_ while grey bars indicate the intersection between si and GSN_∼mito_.

The predictor iPSORT(s2) shares the least similarity to the other four predictors (s1, s3, s5, and s8). iPSORT (s2) holds 2,007 proteins, representing >40% of its total prediction set (4,972 proteins), which are not predicted to be mitochondrial by any other programs. By contrast, the predictive set by Predotar (s5) had the highest shared proteins with the other predictors, with only 35 proteins (3% of the total Predotar set) predicted by this program alone. To evaluate the consistency, we calculated the proteins uniquely predicted not to be localized to mitochondrion by a given program while predicted to be as mitochondrial localization by the other four programs. WoLF PSORT predictor (s8) displays a significant uniqueness in its prediction inventory, with 360 proteins uniquely predicted not to be mitochondrial, whereas the other four programs were relatively in agreement. Totally, 8,158 predicted proteins are designated to mitochondria by combined results from those five programs and meanwhile, 615 proteins are shared by the four commonly used predictor programs (i.e. TargetP, MitoProtII, iPSORT and Predotar) and all are included in ArathMitoP set. Similar relational searches were also investigated previously [60], but their analysis generates an enriched mitochondrial inventory that excluded many real mitochondrial proteins with genuine evidence from experiments (GSP_mito_). Therefore, it shows that only one or two programs can not substantially conclude the proteins that are likely to be mitochondrial destination, because each bioinformatics method can highly influence the predictive set. Besides these comparisons, direct overlaps among the nine predictors are shown in Fig. 2. The correlations of each two predictors are also computed for evaluating their independences.

Complementarily, we also made use of *Arabidopsis* homologs of the ancestry and orthologs of eukaryotic mitochondrial proteins and, found that phylogenetical clues have certain limitation for inferring protein localization. Previous researchers analyzed proteins' localization in a cell according to their phylogenetic profiles. Application of the method reveals that nucleus-encoded proteins previously known to be destined for mitochondria fall into three groups: prokaryotic-derived, eukaryotic-derived, and organism specific [61]. It has been suggested that a large proportion of mitochondrial proteins evolved from prokaryotic lineages (50 to 60%), with the remaining proteins consisting of an eukaryotic subset (20 to 30%) and a speculative species-specific subset (20%). Here, we utilized ancestry and eukaryotic mitochondrial proteins of model species extracted from SwissProt as reference datasets and transferred the protein localizing annotations through the orthologous or homologous relationships to *Arabidopsis* proteins. A total of 1,960 *Arabidopsis* mitochondrial proteins were identified as putative homologs of 834 proteins encoded by *R. prowazekii* 1.1-MB genomes. Similar comparisons with human, mouse and yeast mitochondrial proteome identified 659 putative orthologs to human mitochondrial proteins, 705 putative orthologs to mouse mitochondrial proteins and 652 putative orthologs to yeast mitochondrial proteins within *Arabidopsis*. The putative orthologs derived from these four comparisons has a strict common overlap of 144 proteins, 91 of which are included in ArathMitoP set. False discovery rate of Ancestry homologs predictor (s10) archives 36.12% in accordance with the previous estimation that 50% or more of the genes that encode for the modern mitochondrial proteome originated directly from the host nuclear genome by the duplication and divergence of existing genetic material, rather than indirectly through gene transfer from the endosymbiont genome [62].

Through high-throughput microarray technique, we can monitor gene coexpression trends across various conditions, treatments, or samples from abundant gene expression profiles (Data S3) because different subcellular compartments often show distinct subcellular environments and proteins found within the same localization play similar function roles on time synchronization [57]. A compendium of gene-expression datasets were extracted from AtGenExpress, including 459 biosamples and 1,027 slides on Affymetrix platform (ATH1) (based on Feb. 20^th^, 2007) for the whole *Arabidopsis* genomes. Through classification training on such comprehensive gene-expression profiles by using the decision tree method (Materials and Methods), we successfully predicted 54.09% of known *Arabidopsis* mitochondrial proteins and 1,291 potential novel mitochondrial proteins with 5.22% false discovery rate achieved by training on the GSP and GSN via the decision tree classifier with 10-fold cross-validations.

Finally, we used ROC curves to illustrate the predictive power of individual approach and Bayesian Network integration performance (Materials and Methods). A good feature with high predictive power should have a large number of true positives and a small number of false positives simultaneously. In this case, the ROC curve climbs rapidly away from the origin (lower left hand corner of the graph). The steeper the slope of ROC curves is, the better the approach is. There exist prominent differences between the features in terms of the comparison with the positive and negative gold-standard datasets. Through multi-predictors' integration, our synthesized ArathMitoP set properly includes most of known mitochondrial proteins (61.41%) than other predictors with less FDR (6.95%) (**Fig. 1A**). Obviously, the benefit of genomic-wide integration is the substantial improvement in coverage of true positives and the decrease of false positives. Alternative predictions from minified integration were also evaluated (Fig. 1B–D), but it has limited accuracy and sensitivity. Thus, we chose the better predictions from ArathMitoP set that expends the catalog of nucleus-encoded mitochondrial proteins to 2,311 with relatively high confidence on the same evaluation framework (e.g., GSPmito and GSN∼mto). Moreover, by joining experimentally verified ones with ArathMitoP and excluding false positives, we got a set of 2,585 nonredundant mitochondrial proteins, named CoreMitoP, including 456 proteins identified by mass spectrometry and 615 overlapped proteins from four bioinformatical predictors (s1, s2, s3, and s5) (**Data S4**). Such CoreMitoP set can be queried from our Arabidopsis protein interaction database (http://www.megabionet.org/atpid/webfile/) [55] and provides rich information to facilitate the researches for better understanding of mitochondrial functions.

### The Selection of LR Threshold and Cross-validation

Here, the threshold of ArathMitoP LR (Likelihood Ratio) is set at 1.37, so that the integrated predictions achieve the least false positives rate (2.53%) and the largest coverage of true predictions (84.67%) based on the comparison with GSN_∼mito_ and GSP_mito_ at the same time (Fig. 1, and Fig. 2). Interestingly, determining the prior odds O_prior_ is somehow arbitrary that it requires an assumption about the number of positives (mitochondrial proteins). Meanwhile, experimental approaches have also sought to define the size of the mitochondrial proteome. Two-dimensional electrophoresis studies indicate that mitochondrial samples from plants can be resolved into 500 to 1,500 protein spots [21], [22], [63], [64]. Based on previous estimation, we considered that ∼1,500 positives is a conservative lower bound for the number of mitochondrial proteins (or gold standard positives). Given that there are approximately 30,480 nucleus-genome encoded proteins in total, the prior odds would then be about 1 in 19, we have set the summed log LR >1.27 to guarantee 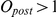 (See **Method and Materials**). Therefore, logLRo = 1.37 as threshold may effectively provide better selection for ArathMitoP as shown on ROC curve (Fig. 1A and Fig. 1B).

To assess the robustness of Bayes integration, we carried out 5-fold and 10-fold cross-validation (5CV and 10CV) test for Bayes integration. Firstly, we constructed training and testing datasets from original positive and negative gold-standard datasets with Bayes integrated log LR. The gold-standard positives and negatives are divided into N equally sized parts. Then, each of the (N-1) sets was used as training set and aside another part as a testing set. Consequently, 5CV got 75.7% specificity, 91.4% specificity and 85.5% accuracy; 10CV got 75.7% specificity, 91.9% specificity and 85.8% accuracy. The results demonstrate that Bayes network for mitochondrial protein integration is robust.

### Functionality Enrichment of *Arabidopsis* Mitochondrial proteomics

**Figure 3:**
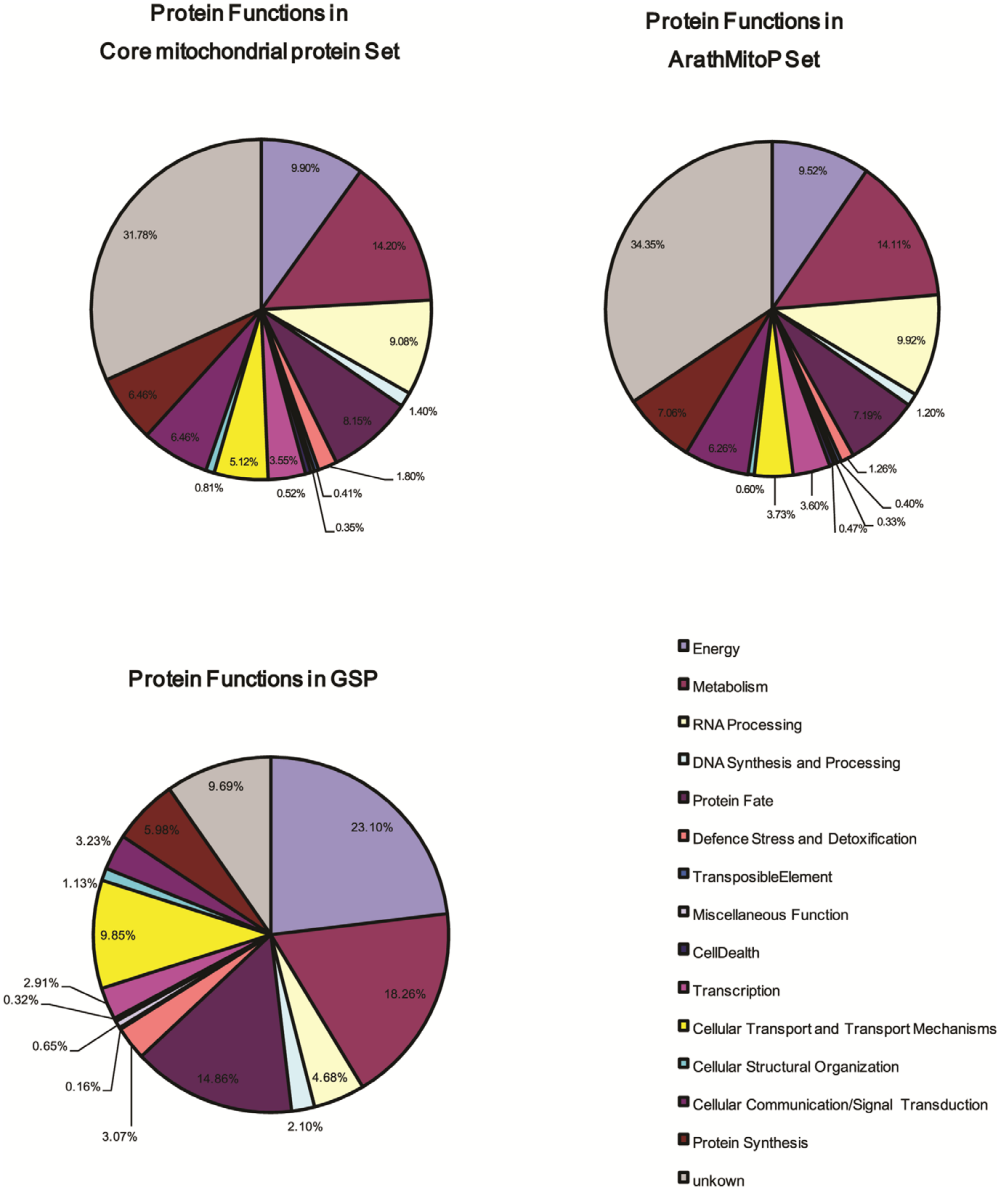
The enrichments of major functional categories for proteins generated from the fourteen genome-wide predictors (s1–s14). Fourteen major Gene Ontology categories were assigned to each genome-wide predictor and each functional category' enrichment for each predictor is shown in different colored bars. Grey bars indicate the proteins with unknown functions. Because of the relative quantity of predictors from s1–s9 generated by bioinformatics tools are large containing thousands of proteins, the percentage of unknown proteins is larger than that of other predictors. Predictors using homolog or orthologs methods have little unknown proteins. Meanwhile metabolism, energy, protein synthesis, transport functional categories are enriched in all predictors. However, DNA Synthesis and Processing are enriched only in predictors of s1∼s9, s13 and s14. Signaling transductions and cellular communications are not enriched in predictors like s11, s12, and s13, but enriched in other predictors.

**Figure 4:**
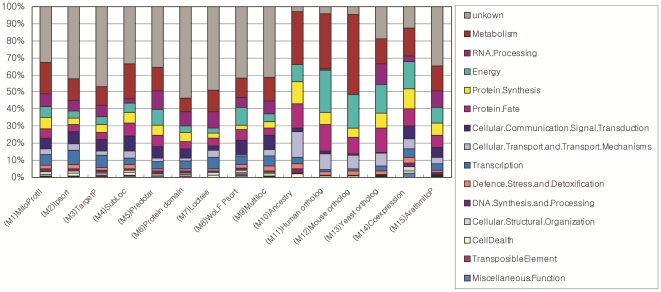
Major function categories of proteins within ArathMitoP Set, CoreMitoP and GSPmito. Twelve protein functional divisions are considered and used for function assignment to proteins in ArathMitoP, CoreMitoP and GSPmito with Gene Ontology annotation. Several major functions exerted by mitochondria include 1) cellular Communication/Signal Transduction, 2) Cellular Structural Organization, 3) Cellular Transport and Transport Mechanisms, 4) Defense stress and detoxification, 5) DNA Synthesis and Processing, 6) Energy, 7) Metabolism, 8) Miscellaneous Function, 9) Protein Fate, 10) Protein Synthesis, 11)RNA Processing, 12) Transcription and unclassified ones.

CoreMitoP is more reliable and complete dataset for mitochondrial proteome currently. We categorized their biological function by using Gene Ontology annotation. Most of proteins in CoreMitoP have been annotated with relative functions, while other 559 proteins are completely novel. we can classify all proteins in CoreMitoP into broad functional divisions (**Figure 2** and **Data S5**) referring to *Arabidopsis* annotations from SUBA Database [29]and Gene Ontology. Most of these involve mainly in several functional groups, such as energy (6.57%), metabolism (9.43%), RNA processing (6.03%), protein fate (5.42%), protein synthesis (4.29%), cellular communication/signal transduction (4.29%), cellular transport/transport mechanisms (3.40%) and transcription (2.36%), In addition, 31 proteins involve in the process of defense stress and detoxification and 14 proteins act in cellular structure organization. Other 9 proteins participate in cell death and 6 proteins are relative to miscellaneous functions. The function categories for individual predictors (s1∼s14) are shown in Fig. 3. Metabolisms, transport, protein fate and protein synthesis each occupy large portions of protein functions with similar percentage. Meanwhile, the composition of function categories in ArathMitoP set and GSP dataset are also illustrated in Fig. 4.

### Protein function inferring from Protein interaction network

ArathMitoP set contains 2,311 integrated mitochondrial proteins from fourteen genome-wide predictors and the assembled CoreMitoP contains 2,585 potential mitochondrial proteins. We assigned 14 function categories defined by Gene Ontology to each protein in ArathMitoP set and CoreMitoP set, respectively. However, 559 proteins in CoreMitoP have no any clear functionality yet (**Data S5**). In an attempt to resolve this issue, we conducted protein functionality prediction by evaluating the similarity of topological properties between the protein and its level-1(direct) or level-2 (indirect) neighbors in protein interaction networks (PIN). Since the characters of the genes and cellular events exerted by protein function mostly depends on the underlying networks in form of protein interactions, and protein functionality can be deduced from the relationships between interactors and characters of their topological structure of the PIN (Materials and Methods).

New functionalities for predicted proteins were assigned by transfers of functions from its neighborhood according to Gene Ontology (GO) annotations. It provides an alternative avenue of systematical discovery of protein functions from innovative molecular network perspective. Originally, a subnetwork consisting of CoreMitoP proteins was reconstructed from global *Arabidopsis* PIN. Then, 14 different functional categories have been assigned to each protein in the mitochondrial PIN. Meanwhile, 28,091 reliable *Arabidopsis* protein–protein interaction pairs by our previous research were available from AtPID [55]. The Arabidopsis PIN consists of 24,418 predictive PIN pairs generated from ortholog interactome, microarray profiles coexpression, GO annotation enrichment, and conserved domain and genome contexts like gene fusion method. The rest 4,695 pairs with 1,875 proteins involved were manually curated from the literatures and databases (e.g. BIND, InAct and TAIR), as well as other 800 pairs reconstructed from enzyme complexes in KEGG. Otherwise, protein function categories adopted here are from Gene Ontology annotation for *Arabidopsis*. Particularly, we focused on the 559 proteins in CoreMitoP that have no any clear biological functions. In the principle of network-based search for protein function, in fact, only part of interested proteins in CoreMitoP proteins can be deduced with multiple significant functionalities from global *Arabidopsis* PIN.

As expected, according to FunScore cutoff ( = 0.03) that was trained from other known protein-function pairs (**Data S6**), we preliminarily choose 425 more reliable function categories for 148 mitochondrial proteins within CoreMitoP. After 10,000 random permutations for *Arabidopsis* functional categories to obtain null distribution for each protein-function FunScore, we gained 416 significant potential functions (p<0.05) for the 148 proteins, covering 26.7% of total 559 unknown core mitochondrial proteins (**Data S7**).

### Mitochondrial stress response in *Arabidopsis*

In past two decades, the knowledge about responses of plant *Arabidopsis* to environmental stresses has been accumulated dramatically. Confirmatively, mitochondria tightly contribute to this process. Besides cell-specific and treatment-specific studies by mutagenesis strategy, to identify genes of potential importance to stresses stimulates investigators to utilize high-throughput techniques of global expression profiling which can reveal transcriptional changes on a genome-wide scale [65]. A global overview of mitochondria responses to stress has been reported [66]. Since the dynamics of proteins can be observed in multiple aspects, such as various cellular localizations in spatial scale, transcriptional changes in gene expression level, and post-transcriptional mechanisms or protein-protein interaction forms, we indeed require understanding a plant's response to a stress with comprehensive evaluation of stress-induced changes in gene expression and also need to capture the relationships of proteins or its genes and well-organized functional modules or pathways of them involved in the responses.

In an attempt to explore the behavior of mitochondria in response to stress, 2,649 induced or repressed genes showing greater than 2-fold change in response to salt, cold, osmotic and draught stresses were collected from previous study [67] (**Data S8**). Using global *Arabidopsis* 28,091 PINs, we reconstructed a stress protein interaction network, named SPIN, which containing 6,891 interaction pairs involving 4,823 proteins (**Data S9**). There are 1,497 stress-related proteins/genes and total 503 defined core mitochondrial proteins participating in the SPIN. In particular, 147 mitochondrial proteins represent differential changes on gene expression level (**Data S10**) and their functionalities are annotated from Gene Ontology (**Data S11**). We can observe that mitochondrial proteins (blue) are interweaved with other proteins coming from other localizations in response to stress. It suggests that more dynamical communications and transportations exist among mitochondria and other organelles.

We assessed the overrepresentation of GO categories in SPIN and obtained several functional modules that correlating with plant stress responses with self-consistency (**Data S12**). Hypergeometric test and multiple testing corrections using Benjamini&Hochberg (FDR) approach was conducted by BINGO plugin [68] embeded in Cytoscape software [69]. In SPIN, many functional modules act in responses to abiotic stimulus (pval = 2.36e–54), oxidative stress (pval = 8.76e–41), osmotic stress (pval = 3.50e–15), cold (pval = 7.07e–13), salt stress (pval = 2.23e–12), and water deprivation (pval = 3.49e–11) (**Data S12**). In plant cells, calcium plays roles as a universal transducer coupling a wide range of extracellular stimuli with intracellular responses and abundant MAPK signaling cascades [70] [71], [72]. Different extracellular stimuli trigger specific calcium receptors. SOS pathway functioning in response to calcium- and salt-stress signaling in plants might have general implications and plays important role in plant growth and development. Calcium permeable ion channels, Ca2+/H+ antiporters and Ca2+-ATPases, are responsible for drought stress signal transduction directly or indirectly. Some proteins of CoreMitoP in SPIN show response to cadmium ion, such as NTRA, ASP1, GDH2, CAT3, etc. GLY3, GDH2, and STRS2 etc., also function in response to salt tolerance (Data S9). Several proteins of CoreMitoP participate in ATP binding with ATPase activity, like BCS1, NFS1, CLPX, etc. Otherwise, the stresses also affect the cellular gene expression machinery and it is possible that molecules involved in nucleic acid metabolism including helicases are likely to be affected [73]. PMH1, STRS2, PMH1 and other newly predicted ones (e.g., AT1G02370, AT1G61640, AT3G18970, and AT2G27800) act in ATP-dependent helicase activity and others from core mitochondrial set like MYB28 and SCA3 behavior transcription factor activities. Meanwhile, AT2G27330 related to nucleic acid binding and AT5G65360 correlated with nucleosome assembly suggests that the active transcriptional events will occur during the stress response.

Furthermore, protein phosphorylation in mitogen-activated protein kinase (MAPK) pathways transfers signal from sensors to exert significance to plant stress tolerance. MAPK cascade minimally consists of a MAPKKK– MAPKK–MAPK module that is linked in various ways to upstream receptors and downstream targets [74]. Several functionalities of CoreMitoP proteins inferred from network correlates with kinase activity and protein amino acid phosphorylation and these proteins may act in this sort of processes. Specifically, PUMP1 participates in oxidative phosphorylation uncoupler activity and AT2G18890 involves in protein amino acid phosphorylation as previous reported. (**Data S7**).

Besides protein phosphorylation and transcription, other post-translational modification like ubiquitination regulates the activation of pre-existing molecules to ensure a prompt response to stress. As we have known, in stress-induced ethylene signaling pathway, CTR signaling cascades and joint kinase cascases, the downstream regulator, EIN3, accomplishes stability by F-box–containing proteins that participate in the formation of a SKP1/cullin/F-box complex that targets proteins for degradation by the proteasome [75]. 3 CoreMito proteins predicted from PIN may involve in the formation of ubiquitin ligase_complex. Two of them, AT1G52620 and *AT5G02860*, are both characterized by pentatricopeptide repeat (PPR) tandem of a degenerate 35 amino acid motif. Most of PPR proteins have roles in mitochondria or plastid [76]. Some of these proteins have been shown to play a role in post-transcriptional processes within organelles and they are thought to be sequence-specific RNA-binding proteins [77], [78], [79]. Another CoreMito protein, AT5G02700, contains cyclin-like F-box domain. The F-box domain was first described as a sequence motif found in cyclin-F that interacts with the protein SKP1 [80], [81]. This relatively conserved structural motif is present in numerous proteins and serves as a link between a target protein and an ubiquitin-conjugating enzyme. The SCF complex (e.g., Skp1-Cullin-F-box) plays a similar role as an E3 ligase in the ubiquitin protein degradation pathway [82], [83]. Different F-box proteins as a part of SCF complex recruit particular substrates for ubiquitination through specific protein-protein interaction domains.

Additionally, cross-connections exist among diverse signaling pathways, clearly demonstrating further and superimposed complexity levels in the response to environmental changes [72]. Besides in response to salt, cold and draught/osmotic stress, we found that many mitochondria proteins in SPIN versatilely act in functions of apperceiving extracellular stimulus (e.g., jasmonic acid(JA), cytokinin, auxin, abscisic acid (ABA), ethylene, and salicylic acid(SA)), bacterium, fungus and incompatible interaction, heat, hypoxia, oxidative_stress, wounding, blue light/red or far red light. Practically, the reactions in plant to salt stress may trigger diverse signaling pathways and biosynthesis pathways, such as ABA signaling, JA synthesis and signaling pathways, as well as auxin and SA reactions. The cross-talks interweave and constitute the complicated plant response mechanism to stresses. All subnetworks of SPIN in response to various abiotic and biotic stresses are listed in Data S10 with the participated components. The discovery of novel genes function also reveals the diversity of mitochondrial protein functions about plant's adaptation in stress environment. PIN-based approach indeed provides the basis of effective engineering strategies leading to our clear understanding of their roles in stress.

## Discussion

### Correlation and statistical dependence among fourteen Genome-wide predictors

Since we implement Narive Bayes Network to integrate fourteen genome-wide predictors, it's necessary to investigated whether the correlation and dependencies existed among predictors. We firstly calculated the Pearson correlation coefficients (CCs) [84] between each two predictors (Table 3). None of the features exhibit significant large correlation, except for the situations for several predictors from the phylogenetic approaches (s11–s13). There is a small correlation scale (corr = 0.01∼0.42) among predictors s1–s9, while a larger correlation scale (corr. = 0.20∼0.66) among predictors s10–s13. Predictor s14 from co-expression method has a smaller correlation scale (corr. = 0.03∼0.16) with all other predictors. As expected, some of the 14 predictors used similar algorithms or pattern information, From s1 to s9, the predictors were trained or based on the amino acid features; the methods of s10 to s13 are based on conserved sequence features or orthologous mapping.

**Table 3:** Pearson correlation coefficients and overlaps between genomic predictors.

Overlap (b)CC (a)	s1	s2	s3	s4	s5	s6	s7	s8	s9	s10	s11	s12	s13	s14
(s1)MitoProtII	1	2077	1577	893	928	986	772	641	1567	658	302	295	322	458
(s2)Ipsort	0.3624	1	1981	880	912	1121	897	629	1708	547	274	228	297	433
(s3)TargetP	0.3575	0.429	1	626	872	964	635	608	1486	379	228	197	235	323
(s4)SubLoc	0.1138	0.0793	0.0819	1	312	564	564	274	884	419	234	251	225	344
(s5)Predotar	0.387	0.3417	0.4273	0.093	1	430	328	361	734	236	181	151	187	215
(s6)Protein domain	0.2163	0.2254	0.2688	0.091	0.2072	1	527	336	839	226	120	104	140	217
(s7)Loctree	0.1014	0.1081	0.1048	0.0554	0.1159	0.0959	1	218	723	235	157	202	191	244
(s8)WoLF Psort	0.2632	0.2282	0.2982	0.0835	0.3084	0.1621	0.0641	1	508	142	113	103	104	157
(s9)Multiloc	0.3452	0.3408	0.3995	0.1593	0.3425	0.2148	0.1294	0.2338	1	349	207	242	241	307
(s10)Ancestry	0.154	0.0874	0.0805	0.0764	0.1167	0.0315	0.0137	0.0579	0.0637	1	313	245	259	330
(s11)Human ortholog	0.1399	0.1043	0.1196	0.1069	0.1868	0.0532	0.064	0.114	0.1012	0.2502	1	459	305	201
(s12)Mouse ortholog	0.1272	0.0697	0.0905	0.1111	0.1445	0.0362	0.0901	0.0965	0.1191	0.1792	0.6663	1	253	183
(s13)Yeast ortholog	0.1543	0.1196	0.126	0.1019	0.1953	0.0701	0.0896	0.1036	0.127	0.2021	0.4541	0.3596	1	190
(s14)Coexpression	0.0942	0.0631	0.0702	0.0606	0.1144	0.0385	0.0296	0.0791	0.0593	0.1294	0.1612	0.1366	0.1516	1

a Pearson Correlation coefficients.

b overlaps between the two genome-wide predictors.

Calculated correlation coefficients between genomic predictors are used to overview the statistical dependence that is required for Naïve Bayes integration. The direct comparison and overlaps among predictors reveal the similarity of the predictions performed by such 14 genome-wide predictors.

Alternatively, direct comparisons between each two predictors were performed for obvious sense of similarity of each two methods in results. We noticed that there may exist contradiction that the independency among diverse predictors required by the Naïve Bayesian approach may cause less consistency among various predictors. However, through Bayes integration procedure, complementary evidences across various systems or predictors can possibly meet the common sense of researchers because Bayes Network can mostly extended the predictions on one benchmark applied by relatively large sets of GSP and GSN.

### Phylogenetic evidence of the mitochondrial proteome

This is fundamental and prerequisite for understanding the role of organelles through comparative analysis with the functions of the same organelles in other species. Nucleus-encoded proteins destined for different subcellular locations have measurable distinct phylogenetic distributions of homologs that can be described with a phylogenetic profile that specifies the pattern of occurrence of a given protein among completed sequenced organisms. This allows the BLAST-based transfer of annotation.

Based on genome contexts of various species, proteins pertaining to mitochondria have been identified. The common set of the four features (s10–s13) are only skewed towards to the function category of energy, with some members from the metabolism and protein fate categories (**Fig. 3**). However, a few putative proteins are classified into mitochondria-cellular interaction (transcription, DNA synthesis and processing and RNA binding/processing). Thus, although the major functions of mitochondria are conserved, mitochondria may recruit novel proteins to play roles in the communication and regulation between the mitochondrion and intracellular environment.

### Integration of broad microarray data resources for mitochondrial protein prediction

Gene-expression profiling has historically been applied to elucidate the mechanisms underlying biological pathways and to reveal protein subcellular compartments. Many gene-expression experiments envisage their usage as means to catalog the biological responses to a large number of diverse perturbations. We hypothesized that perturbations in plant cells might also provide an approach that reveals protein spatial properties systematically and biologically. Conceivably, a large number of variables would need to be considered, including cell lines, tissue, concentration, and treatment duration. In plant, previous researches have shown that mitochondrial functions coordinated with other organelles in the cell can be elucidated by several potential modes, such as retrograde regulation (refers to the regulation of nuclear gene expression by metabolic changes or signals originating in the organelle) that plays a role in controlling synthesis of mitochondrial proteins in plants, and common transcription responses to external stimuli. Therefore, besides microarrays under normal conditions, gene expression profiles focusing on stress time courses, including cold ,osmotic, drought, heat, salt, wounding and oxidative stress, can also be very informative for the mitochondrial protein identification. Chemical compounds (e.g. ABA, ACC, IAA, Methyl Jasmonate, GA3 and Cytokinin et al.) and environmental stimuli (e.g., UV-B and light treatments) screens can probably also profile a subset of functions related to mitochondrial proteins.

Meanwhile, one would generate profiles in a wide diversity of established genotypes. So, we pursue to take consideration of diverse mutants for compensation. They can be partial evidence to infer mitochondrial proteome through gene coexpression N50 metric here (Materials and Methods). Especially for various mutants, we expect that even if knock up/down or mutant genes can influence normal gene expression profiles, the proteins affected by them may still involve in similar stringent pathways and hold similar co-expression trends or patterns. Thus, expression changes of the genes in response to the stimuli (/mutants) can also be used to infer gene effects through the simultaneous adjustments of other genes and may be detected by using conventional measures of correlation, such as the Pearson correlation coefficient.

Additionally, the 725 proteins in the CoreMitoP list for which mass spectrometry evidence exists for subcellular location, about 500 are found in mitochondria and 256 are experimentally found elsewhere (ranging from plastid to nuclear to Golgi and plasma membrane), hence only 62% of these proteins are mitochondrial. The situation is similar if we consider GFP image evidence for location, there are 226 of the CoreMitoP set for which GFP data are available, 150 are in mitochondria, and 73 are confirmed in another location. However, the protein dynamics have already convinced us that most mitochondrial proteins have multiple compartment targets, besides mitochondria, in available GFP and MS supports in training dataset (GSPmito). So, the training set remains the potential multiple-target pattern for predicting. Even though current experiments have no supports for such predictions targeting to mitochondria now, that won't be proper to lead to the conclusion that there doesn't exist multiple targets for such proteins because we may disregard the dynamic properties of them. Otherwise, we have noticed that mitochondrial proteins actively relate to proteins from other organelles. Hence, it implicates that the proteins dynamics make the protein fate and subcellular target complicated and the experimental evidence supports has limited power to validate the computed predictions.

### The predictive power of network-based protein function annotation

Besides predicting core mitochondrial proteins (CoreMitoP), unknown proteins have also been investigated, as well as other CoreMitoP proteins with current known functions, based on PIN. The network-based strategy for inferring protein functions does not rely on traditional ways, like gene sequence or protein structure homologies, and it can be applied to any organism and a wide variety of experimental data sets with the vast accumulating resources of PIN for model species. So, it may provide a novel perspective for protein function prediction from statistically.

In fact, the predictive power of network-based protein function annotation is restricted to the quality and quantity of the reliable *Arabidopsis* PIN. Currently, 51.01% of 2,686 CoreMitoP proteins have interaction records in AtPID. In details, the abundance of latest Gene Ontology version for *Arabidopsis* on level–5 and level–4 coverage 58.53% and 57.61% proteins in *Arabidopsis* PIN, respectively. Hence, it is reasonable to obtain limited power for predicting unknown functions for CoreMitoP proteins labeled by grey color in mitochondrial PIN. We believe that future resources for reconstructing PIN will facilitate post-genome research more, especially about protein functions analysis.

### Conclusions

Subcellular proteomics is an attractive way of presaging complex systems in biology. Understanding the subcellular compartment in which a protein is likely to reside can facilitate the proteomics analysis and protein isolation experiments. In this study, we have obtained a reliable set of mitochondrial proteins encoded in *Arabidopsis* nuclear genome by using an integrative approach. In contrast to previous methods that rely on target sequence properties, we applied additional clues from broad co-expression profiles, ancestry homologs and eukaryotic orthologs and improved the predictive performances for deducing mitochondrial proteins, although systematic research on mitochondrial subcellular location is not first proposed here and many algorithms have been deliberately devised for this task. More precise predictions and assessment for *Arabidopsis* mitochondrial proteomics from diverse methodologies or datasets have not been considered yet. In this study, Bayesian Network has also assess the large dispersed datasets under the same probabilistic benchmark, in which each genome-wide predictor can individually contribute to the total integration power. The CoreMitoP we defined, by combining verified mitochondrial proteins with ArathMitoP set and excluding false positives provides more reliable and comprehensive catalog of mitochondrial proteome. Besides the current function annotation to CoreMitoP proteins, we exploited *Arabidopsis* protein interaction network to assign functionality to unknown proteins, through considering its neighbors and position in topological structures. Then, mitochondrial functions in stress responses are queried and related functional modules in SPIN convinced us that CoreMitoP proteins participate in multiple stress responses, besides salt, cold and drought. Newly predicted functionalities for CoreMitoP based on network (PIN) have large relevance to diverse cross-talks among signaling transduction, transcriptional changes and post-reputational controlling in response to environmental changes. We hope statistical integration and systematical inferring about protein function facilitate the discovery of molecular mechanisms related to mitochondria.

## Supporting Information

Data S1 **Likelihood Ratio (LR) table for all Arabidopsis proteins and integration process using 14 predictors.** This file contains a table, named as ArathMitoP_LR, which includes each predictor's LRs evaluated using GSPmito and GSN∼mito training data (shown in s1∼s14 columns), and the final integrated LRs for Arabidopsis proteins are listed in LR Column.
(XLS)

Data S2 **AtGenExpress microarray profiles for co-expression inferring.** Twenty-four datasets containing 1,027 AtGenExpress microarray profiles were used for the analysis of gene co-expression that as a predictor s14.
(XLS)

Data S3 **Gold-standard positives (GSPmito) and gold-standard negatives (GSN∼mito) information.** This file contains GSP**mito** dataset and GSN∼**mito** dataset. Gold-standard positives (GSP**mito**) were generated from five experimental sets, including AmiGO, GFP assay, MS_MS assay, TAIR, and UniProt. Meanwhile, Gold-standard non-mitochondrial proteins GSN∼**mito** was generated from SwissProt, including proteins localized at cell plate, cytoskeleton, cytosol, endoplasmic reticulum, extra cellular, Golgi apparatus, nucleus, peroxisome, plasma membrane, plastid and vacuole. All original downloaded and collected data sets have been verified through TAIR annotation manually.
(XLS)

Data S4 **Protein list of ArathMitoP set and CoreMitoP.** The proteins of ArathMitoP set and the newly integrative **CoreMitoP** proteins defined in this paper with high confidence through the integration procedure are listed in this file.
(XLS)

Data S5 **CoreMitoP protein functions annotated by using twelve functional categories.** A table listing the Arabidopsis **CoreMitoP** proteins in twelve functional divisions separately, according to the SUBA database collections.
(XLS)

Data S6 **FunScores for validation datasets.** So called Validation datasets are the protein-function pairs those having been annotated by Gene Ontology. In attempt to choose proper FunScore computed by network-based method and filter out less reliable protein-function pairs, we inquired the FunScore of validation pairs and used 0.03 as preliminary threshold. All validation pairs and their FunScores are listed in this file.
(XLS)

Data S7 **Significant functionalities for the unknown CoreMitoP proteins.** Based on network protein function analysis and using randomizations for functional categories, significance of each filtered functionality for unknown CoreMitoP proteins is individually evaluated and corresponding p value and functional categories are listed in this table.
(XLS)

Data S8 **Differentially expressed genes of** ***Arabidopsis*** **in response to salt, cold and osmotic/draught stresses.**
(XLS)

Data S9 **Arabidopsis Stress response protein interaction network (SPIN).**
(XLS)

Data S10 **CoreMitoP proteins involved in SPIN.**
(XLS)

Data S11 **Functional annotation of CoreMitoP proteins involved in SPIN.**
(XLS)

Data S12 **Significant subnetworks from Arabidopsis SPIN in response to stress tolerance.** 42 significant sub-modules and networks related to diverse stress responses are detected. Reconstructed modules are illustrated in figures.
(XLS)
